# Results from the “Me & My Heart” (eMocial) Study: a Randomized Evaluation of a New Smartphone-Based Support Tool to Increase Therapy Adherence of Patients with Acute Coronary Syndrome

**DOI:** 10.1007/s10557-022-07331-1

**Published:** 2022-04-20

**Authors:** Florian Krackhardt, Magnus Jörnten-Karlsson, Matthias Waliszewski, Mikael Knutsson, Anna Niklasson, Karl-Friedrich Appel, Ralf Degenhardt, Alexander Ghanem, Till Köhler, Marc-Alexander Ohlow, Carsten Tschöpe, Heinz Theres, Jürgen vom Dahl, Björn W. Karlson, Lars S. Maier

**Affiliations:** 1 Department of Internal Medicine and Cardiology, Charité Campus Virchow-Klinikum, Charité University Medicine Berlin, Augustenburger Platz 1, 13353 Berlin, Germany; 2 Digital Implementation, Digital Health R&D, AstraZeneca, Gothenburg, Sweden; 3 Late Stage Development, Cardiovascular, Renal, and Metabolism, BioPharmaceuticals R&D, AstraZeneca, Gothenburg, Sweden; 4 Ambulantes Herzzentrum Kassel, Kassel, Germany; 5 Herz-Kreislauf-Zentrum, Rotenburg a. d. Fulda, Germany; 6 Asklepios Klinik St. Georg, Hamburg, Germany; 7 Herzzentrum Wuppertal, Helios-Universitätsklinikum, Wuppertal, Germany; 8 SRH-Waldklinikum, Gera, Germany; 9 Berlin Institute of Health at Charité; BIH Center for Regenerative Therapies (BCRT), Charité-Universitätsmedizin Berlin, Berlin, Germany; 10 Martin Luther Krankenhaus, Berlin, Germany; 11 Kliniken Maria Hilf, Mönchengladbach, Germany; 12 Department of Molecular and Clinical Medicine, Institute of Medicine, Sahlgrenska Academy, University of Gothenburg, Gothenburg, Sweden; 13 Department of Internal Medicine II, Universitätsklinikum Regensburg, Regensburg, Germany

**Keywords:** Acute coronary syndrome, Adherence, Dual antiplatelet therapy, Smartphone-based support, Ticagrelor

## Abstract

**Purpose:**

This study evaluated whether patient support, administered via an electronic device-based app, increased adherence to treatment and lifestyle changes in patients with acute coronary syndrome (ACS) treated with ticagrelor in routine clinical practice.

**Methods:**

Patients (aged ≥ 18 years) with diagnosed ACS treated with ticagrelor co-administered with low-dose acetylsalicylic acid were randomized into an active group (with support tool app for medication intake reminders and motivational messages) and a control group (without support tool app), and observed for 48 weeks (ClinicalTrials.gov Identifier: NCT02615704). Patients were asked to complete the 36-item Short-Form Health Survey (SF-36) and Lifestyle Changes Questionnaire (LSQ), and were assessed for blood pressure and body mass index (BMI) at baseline (visit 1) and at the end of the study (visit 2). Medication adherence was measured using the Brilique Adherence Questionnaire (BAQ).

**Results:**

Patients (*N* = 676) were randomized to an active (*n* = 342) or a control (*n* = 334) group. BAQ data were available for 174 patients in the active group and 174 patients in the control group. Over the 48-week period, mean (standard deviation) adherence for the active and control groups was 96.4% (13.2%) and 91.5% (23.1%), respectively (effect of app intervention, *p* < 0.05). There were no significant differences in blood pressure and BMI between visits. General improvements in SF-36 and LSQ scores were observed for both groups.

**Conclusion:**

The patient support tool app was associated with significant improvements in patient-reported treatment adherence compared with a data collection app alone in patients prescribed ticagrelor for ACS.

**Supplementary Information:**

The online version contains supplementary material available at 10.1007/s10557-022-07331-1.

## Introduction

Ischemic heart disease is a leading cause of morbidity and mortality, resulting in about 9.5 million deaths worldwide each year [1]. In the USA, approximately 1.3 million patients annually are discharged from hospitals following an acute coronary syndrome (ACS) event (ST-segment elevation myocardial infarction [STEMI], non-ST-segment elevation myocardial infarction [NSTEMI], or unstable angina), and patients who survive an ACS event are at high risk of future events and heart failure [2]. To decrease the risk of adverse outcomes following an ACS event, US and European guidelines recommend treatment with a P2Y_12_ inhibitor together with low-dose acetylsalicylic acid (ASA) for up to 12 months, in addition to revascularization, usually by percutaneous coronary intervention (PCI) or by coronary bypass surgery [3–6]. While maintaining adherence to dual antiplatelet therapy (DAPT) is key in patients with ACS, bleeding is a well-known risk with antiplatelet treatment, including gastrointestinal bleeding, as reported by Sarajlic et al. [7]. Behavioral changes that support cardiovascular health include having a good-quality diet (i.e., a Mediterranean diet), sufficient physical activity, not smoking, and having a healthy body mass index (BMI), as well as managing blood cholesterol levels, blood pressure, blood glucose levels, and heart rate [2]. Adherence to preventive treatments and lifestyle changes are key to modify risk factors that affect outcomes after an ACS event, yet persistence with secondary prevention medications post discharge is poor [8, 9]. Patients with a higher risk of acute myocardial infarction (MI) are more likely to be non-adherent than low-risk patients [9]. Treatment non-adherence is associated with increased hospitalizations, poor health outcomes, and high overall healthcare costs [10, 11]. Smartphone-based approaches can offer a cost-effective way to improve adherence [12–15]. Reminders sent via mobile devices were found to increase adherence by 18% in a meta-analysis of randomized clinical trials in adults with chronic diseases [15]. A study by Johnston et al. investigated the effects of an interactive smartphone tool in improving treatment adherence in ticagrelor-treated patients with MI (*N* = 174) over 6 months in a Swedish cohort [13]. While patients self-reported their adherence using the tool, the study also included two follow-up site visits at which quality of life and adherence behavior were assessed (via the Medication Adherence Rating Scale [MARS-5]) and pill counting. Further endpoints included monitored changes in cardiovascular risk factors and patients’ satisfaction with the tool. While the differences were not statistically significant in the study by Johnston et al., increased self-reported drug adherence with improvements in overall health were observed for the active group (with the smartphone tool) [13].

The aim of the current 12-month study was to evaluate if patient support administered via an electronic device-based app increases adherence to treatment and lifestyle changes in patients with ACS treated with ticagrelor as part of DAPT in routine clinical practice. Mobile apps like “Me & My Heart” are a separate device category from Medical Event Monitoring Systems (MEMS) and should not be considered as such.

## Methods

### Study Design

The “Me & My Heart” study (eMocial; ClinicalTrials.gov Identifier: NCT02615704) was an observational, randomized investigation according to paragraph 23b of the German Medical Device Law [16], conducted at 30 study centers in Germany. Patients with ACS were randomized 1:1 to an active group receiving the patient support tool via an electronic device app or a control group receiving an app for data collection only, without the patient support tool. The support tool app has a European Conformity Declaration (Conformité Européene [CE]) mark that identifies it as a class I medical device. In addition, both the active and the control groups were randomized 1:1 to subgroups with or without the use of an electronic tablet dispensing device (MEMS) to detect the date and time of when a tablet is dispensed (twice daily), for evaluation of treatment adherence. The study comprised an initial enrollment and randomization visit (visit 1), a 48-week observation period without on-site visits, and one additional ambulatory visit (visit 2) at the end of the study (i.e., 12 months after the ACS event). The study design and rationale have been reported previously [17].

### Patients

To be eligible for study inclusion, patients needed to be 18 years of age or older and had to have received a diagnosis of ACS (STEMI, NSTEMI, or unstable angina). In addition, eligible patients needed to be receiving treatment with ticagrelor as part of standard clinical practice, with the treating physician intending to continue prescribing twice-daily ticagrelor co-administered with low-dose ASA according to the prescription recommendation within 14 days following the ACS event. The main exclusion criteria were treatment with antiplatelet drugs (other than ticagrelor with ASA), planned thoracic surgery (e.g., coronary artery bypass grafting) or other elective surgery that could not be postponed until after study participation, or any serious/severe comorbidities that might limit life expectancy to less than 1 year. Patients provided written informed consent prior to randomization.

### Interventions

Patients in the active group used the support tool app to enter baseline information and additional data on an ongoing basis, and received individualized feedback including optional daily reminders for medication intake and motivational and informative messages every few days (Supplementary Table 1). Qualitative information on cardiovascular risks in relation to lifestyle choices was displayed graphically throughout the study. Patients in both study groups received self-reporting questionnaires via their apps every 4 weeks to evaluate study endpoints.

### Study Assessments

Key cardiovascular risk factors were assessed by an experienced healthcare practitioner for all patients at baseline (visit 1) and at the end of the study (visit 2), and included blood pressure, BMI, and laboratory measures for levels of low-density lipoprotein (LDL) cholesterol, high-density lipoprotein (HDL) cholesterol, and glycated hemoglobin A1c (HbA1c). In addition, a cardiovascular risk score (GRACE 2.0) was calculated at visit 1 [18]. All patients were prompted via their app to complete the 36-item Short-Form Health Survey (SF-36) and the Lifestyle Changes Questionnaire (LSQ) at visit 1 and visit 2. The SF-36 is a patient-reported, generic, health-related quality of life (HRQoL) instrument that comprises eight subscales: physical functioning, bodily pain, general health, vitality, mental health, and role limitations due to physical, social, and emotional functioning [19, 20]. The LSQ is a patient-reported outcome (PRO) instrument developed specifically for the eMocial study [17]. It comprises questions on adherence to a healthy diet and regular exercise, and on smoking behavior. In addition, all patients were prompted via their app to complete the Brilique Adherence Questionnaire (BAQ) every 4 weeks during the study observation period. The BAQ is a PRO instrument developed for the eMocial study that contains 15 questions (Supplementary Table 2) [17]. With no previously formally validated tool available for measuring adherence, it was necessary to develop an instrument to fit the needs of the study to measure both intentional and unintentional non-adherence. Medication adherence is assessed via questions 1–4 of the BAQ, with question 4 quantifying the number of tablets taken, ranging from 0 to 14 (i.e., one deduction for every missed ticagrelor tablet with twice-daily dosing for the past 7 days): (1) Do you currently take ticagrelor? (2) If not, why are you not taking ticagrelor? (3) Over the past 4 weeks, did you take your ticagrelor tablets every day? (4) For patients who did not take all ticagrelor tablets every day, how many ticagrelor tablets did you take during the last 7 days? Disease understanding and treatment awareness are evaluated based on BAQ questions 5–11, and healthcare utilization is assessed using questions 12–15. App usability was assessed via the system usability scale (SUS) [21], which involves 10 questions to measure subjective system satisfaction using a response scale from 1 (strongly disagree) to 5 (strongly agree) (final score range: 0–100).

### Study Endpoints

The primary endpoint was adherence to prescribed ticagrelor treatment, measured using BAQ questions 1–4, including a score (ranging from 0 to 14) for the number of tablets taken in the previous 7 days. Responses were extrapolated to the previous 4 weeks. Secondary endpoints were as follows: adherence to prescribed ticagrelor treatment, measured using MEMS; changes from baseline to the end of the study in key risk factors (blood pressure, BMI, laboratory measures); change from baseline to the end of the study in HRQoL, assessed using the SF-36; effect on diet, exercise, and smoking behavior, assessed every 4 weeks using the LSQ; and effect on disease understanding and treatment awareness (BAQ questions 5–11) and healthcare utilization (BAQ questions 12–15), assessed every 4 weeks. Exploratory endpoints were as follows: missed tablets (based on MEMS); use of other, non-study medication reminders or health apps; impact of baseline GRACE 2.0 risk score on adherence as measured using the BAQ; and, in the active group, frequency of support tool app usage during the study and app usability.

### Statistical Analysis

For reporting primary endpoint variable data, a dropout rate of 30% was assumed given the real-world setting of the study with no site visits during the observation period, the potential technological barrier in the target population, and the general uncertainty of this novel approach [17]. A sample size of 660 patients was calculated to have 85% power to detect a between-group difference in adherence rate of 7%, assuming a standard deviation (SD) of 25%, using the Student’s *t*-test with a 0.05 two-sided significance level. Patient demographics and clinical characteristics were assessed descriptively. *P* values were calculated with the Student’s *t*-test, paired *t*-test, or chi-square test. Repeated measures analysis of variance (ANOVA) and generalized estimating equation (GEE) modeling were used for analysis of adherence for the primary and secondary endpoints. The repeated measures ANOVA was conducted with the following factors: app (yes/no); MEMS (yes/no); use of alternative medication reminder (yes/no); use of other health apps (yes/no); site; time; GRACE; app and MEMS; and app and time. The primary method for handling missing data was to impute the mean of the values before and after the missing value if this occurred during the study, and to use the last value carried forward if this occurred at the end of the study. In the GEE approach, dichotomized adherence values were analyzed, with values ≥ 90% classified as adherent and values < 90% as non-adherent. Other secondary and exploratory outcomes were assessed descriptively. All statistical analyses were performed using SAS 9.4 (SAS Cary, NC, USA) or SPSS version 23 (IBM, Munich, Germany) statistical software packages.

## Results

### Patients

In total, 676 patients were enrolled and randomized: 342 (50.6%) were randomized to the active group receiving the patient support tool via the app and 334 (49.4%) to the control group receiving an app for data collection only, without the patient support tool. In the active group, 164 patients were randomized to the MEMS subgroup and 178 patients to the non-MEMS subgroup. In the control group, 171 patients were randomized to the MEMS subgroup and 163 to the non-MEMS subgroup (Supplementary Fig. 1). Mean age (active group: 56.6 years; control group: 56.0 years, *p* = 0.472) and sex distribution (male/female, active group: 83.0%/17.0%; control group: 86.5%/13.5%, *p* = 0.213) were similar between the two study groups (Table 1). Likewise, prevalence patterns for concomitant cardiovascular and metabolic disorders, prior PCI, and concomitant medication use did not differ (Table 1). Most patients (active group: 93.6%; control group: 93.1%) were taking at least one other type of cardiovascular medication in addition to ticagrelor at baseline. More than half of patients (active group: 58.8%; control group: 57.4%) reported doing no or only mild regular weekly exercise at baseline without a significant difference between groups (*p* = 0.961). More than one-third of patients (active group: 36.8%; control group: 39.5%) were current smokers at baseline, with no difference between treatment groups (*p* = 0.779). Educational level, living arrangements, and employment status were balanced between the active and control groups (Supplementary Table 3). Overall, 71% of patients (*n* = 483) completed the trial, whereas 29% (*n* = 193) did not. Reasons for not completing included the following: lost to follow-up (*n* = 88); stopped ticagrelor medication (*n* = 50); patient’s decision to withdraw (*n* = 32); incorrect enrollment (*n* = 14) mostly due to an incompatible smartphone, the investigator’s decision (*n* = 1), or other reason (*n* = 1); and death (seven patients died during the course of this study).

**Table 1:** Baseline demographics and clinical characteristics

Variable	Active group (*n* = 342)	Control group (*n* = 334)	*p* value
Age, years, mean (SD)	56.6 (9.1)	56.0 (9.9)	0.472
Male sex, *n* (%)	284 (83.0)	289 (86.5)	0.213
Type of ACS, *n* (%)			
STEMI	130 (38.0)	122 (36.8)	0.903^*^
NSTEMI	165 (48.2)	166 (50.0)	
Unstable angina	47 (13.7)	44 (13.3)	
Unknown status	0 (0.0)	2 (0.6)	
Medical history, *n* (%)			
Hypertension	235 (68.5)	228 (68.3)	0.944
Hyperlipidemia	192 (56.0)	188 (56.3)	0.935
Diabetes mellitus^a^	62 (18.1)	55 (16.5)	0.580
Obesity^b^	82 (23.9)	96 (28.7)	0.153
Prior PCI	202 (58.9)	191 (57.2)	0.653
Co-medication, *n* (%)			
Statin	292 (85.1)	284 (85.0)	0.971
Beta-blocker	282 (82.2)	259 (77.5)	0.129
ACE inhibitor	232 (67.6)	219 (65.6)	0.568
Angiotensin receptor blocker	47 (13.7)	45 (13.5)	0.931
Diuretic	69 (20.1)	59 (17.7)	0.415
Calcium channel blocker	45 (13.1)	39 (11.7)	0.569
Smoking status, *n* (%)			
Current smoker	126 (36.8)	132 (39.5)	0.779^*^
Former smoker	129 (37.7)	115 (34.4)	
Never smoked	78 (22.8)	79 (23.7)	
Unknown status	9 (2.6)	8 (2.4)	
Regular weekly physical activity, *n* (%)			
None	71 (20.8)	64 (19.2)	0.961^*^
Mild	110 (32.2)	107 (32.0)	
Moderate	91 (26.6)	95 (28.4)	
Strenuous	36 (10.5)	32 (9.6)	
Unknown	34 (9.9)	36 (10.8)	

^a^Insulin and non-insulin dependent

^b^Body mass index > 30 kg/m^2^

*p* values were calculated with the Student’s *t*-test

^*^p values calculated with the chi-square test; this was done for type of ACS (STEMI, NSTEMI, unstable angina, unknown status), smoking status (current, former, never, unknown), and regular weekly physical activity (none, mild, moderate, strenuous, unknown)

*ACE* angiotensin-converting enzyme, *ACS* acute coronary syndrome, *NSTEMI* non-ST elevation myocardial infarction, *PCI* percutaneous coronary intervention, *SD* standard deviation, *STEMI* ST elevation myocardial infarction

### Patient-Reported Medication Adherence

Data for the primary endpoint of adherence according to the BAQ during the 48-week observation period were available at all 4-week time points for 174 patients (50.9%) in the active group and 174 patients (52.1%) in the control group. The response rates for the BAQ and LSQ were very similar in the two groups.

**Fig. 1:**
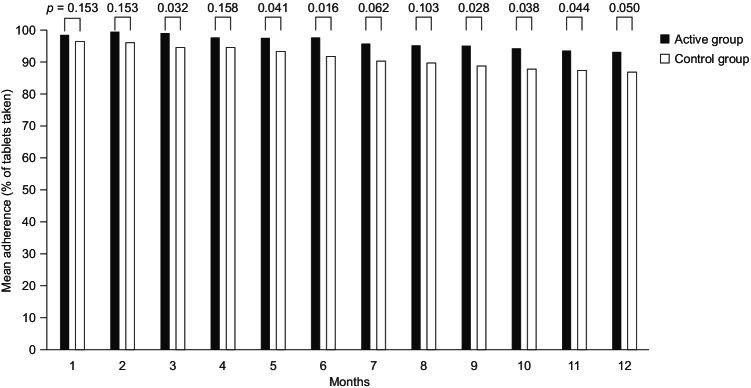
Mean adherence rates, according to the percentage of tablets taken, per 4-week time block in the active and control groups during the 48-week study observation period. Data on adherence from the Brilique Adherence Questionnaire were available for only 174 patients (50.9%) in the active group and 174 patients (52.1%) in the control group

**Fig. 2:**
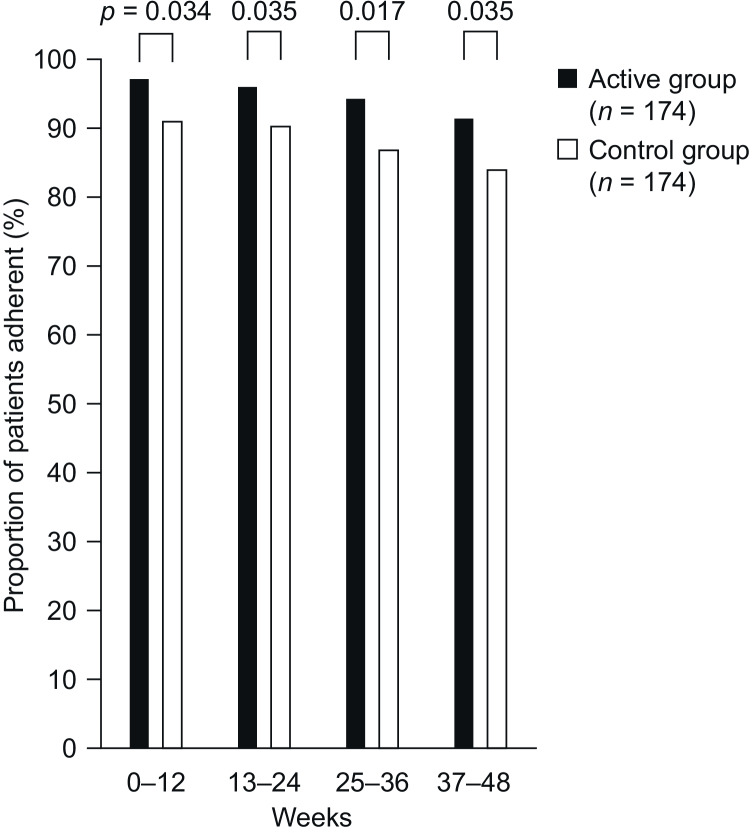
Proportion of patients adherent to treatment, defined as taking at least 90% of their ticagrelor tablets as prescribed. Data on adherence from the Brilique Adherence Questionnaire were available for only 174 patients (50.9%) in the active group and 174 patients (52.1%) in the control group

Figure 1 shows the mean adherence rates, according to the percentage of tablets taken, per 4-week time block in the active and control groups during the 48-week observation period. Among patients with available data (*n* = 348), mean adherence was higher in the active group than in the control group at all time points and declined by the end of the study in both groups. At week 4, mean adherence for the active group compared with the control group was 98.7% and 96.5%, respectively (*p* = 0.153), while at week 48 the mean adherence for the active group compared with the control group was 93.4% and 87.0%, respectively (*p* = 0.05). The mean adherence showed a decline of -5.3% for the active group and -9.5% for the control group. The difference between the two treatment groups became significant for the first time over the first 12 weeks, when patient adherence was higher for active group patients than for control group patients (*p* = 0.032). Over the entire observation period, mean (SD) adherence was 96.4% (13.2%) in the active group and 91.5% (23.1%) in the control group. The repeated measures ANOVA showed a significant effect of the app intervention (active vs control, *p* = 0.014) and time (quarters, *p* < 0.001). Similarly, the GEE analysis showed a significant effect of the intervention (active vs control, *p* = 0.039) and time (quarters, *p* < 0.001). Figure 2 shows the proportion of patients adherent to treatment, defined as taking at least 90% of their ticagrelor tablets as prescribed. As with mean adherence rates, among patients with available data, the proportion of those adherent to treatment was significantly higher in the active group than in the control group from weeks 13 to 48 and declined during the study in both groups, from 97.1% (weeks 1–12) to 91.4% (weeks 37–48) in the active group (-5.7%) and from 92.0% (weeks 1–12) to 83.9% (weeks 37–48) in the control group (-8.1%). No differences were observed by MEMS subgroups or baseline GRACE score (all *p* > 0.2). Data from the individual BAQ questions on adherence (questions 1–4) showed that, among patients with available data, 8.2% in the active group and 11.4% in the control group reported on at least one occasion during the study that they were not currently taking their ticagrelor tablets. Reasons patients provided for not taking their ticagrelor tablets were that this had been advised by their doctor (7 patients; 41.2%), they required a temporary break (e.g., because of surgery, 6 patients; 35.3%), they had switched to another antiplatelet agent (12 patients; 52.2%), or they had decided themselves to stop the medication (2 patients; 8.7%). Among patients with available data, the proportion reporting taking their ticagrelor twice every day during the previous 4 weeks was 94.8% at week 4 and 95.7% at week 48 in the active group, and 95.9% at week 4 and 93.6% at week 48 in the control group. During the study, among patients with available data, 21.7% in the active group and 16.9% in the control group indicated that they had not taken their ticagrelor tablets every day on at least one occasion. The mean (SD) number of tablets taken per week in patients who reported not taking their ticagrelor tablets every day was 12.1 (2.8) in the active group and 10.5 (3.3) in the control group.

### Medication Adherence According to MEMS

**Fig. 3:**
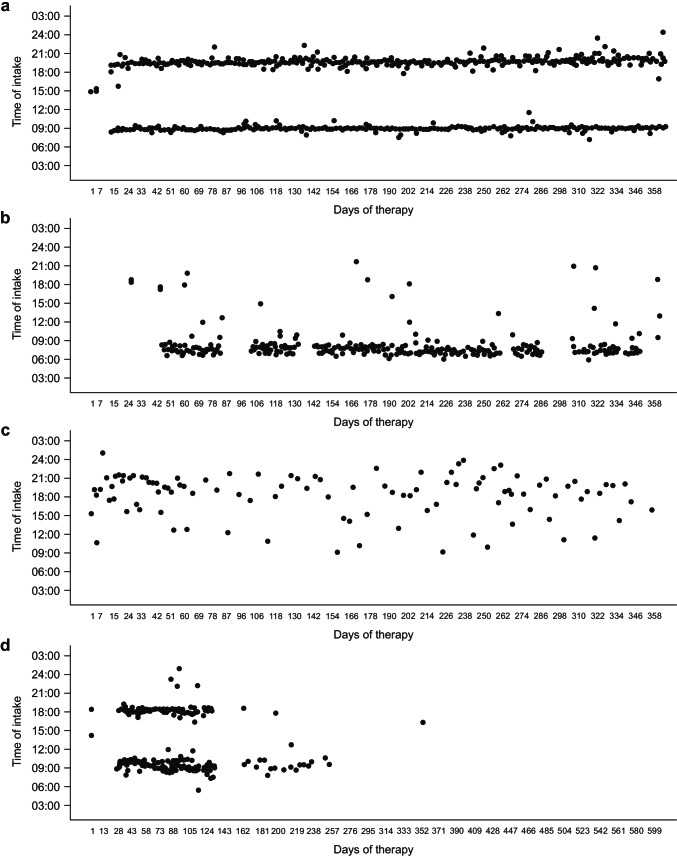
Example dosage pattern data from MEMS for an ideal case (ticagrelor 2 × 90 mg (e.g., at 8 a.m. and 8 p.m.) **a** and semichaotic patterns, including **b** morning adherence and no adherence in the evening, **c** sporadic tablet intake, and **d** an abrupt lack of adherence. Each dot represents a tablet taken. *MEMS*, Medication Event Monitoring System

Of the 316 MEMS devices, 107 contained data (*n* = 48 in the active group and *n* = 59 in the control group). MEMS devices that contained only one medication event (as opposed to two [twice daily]) were excluded (*n* = 6 in the active group and *n* = 12 in the control group). Hence, MEMS data were available from only 42 and 47 patients in the active and control groups, respectively. Independent of the formal analysis for the primary endpoint, it appears to be important to study exemplar dosage patterns for individual patients in the active and the control groups. Statistical analysis was not performed for this data set; however, we report exemplar dosage patterns of four patients who documented their tablet intake over the 48-week monitoring period (Fig. 3). These observed adherence patterns range from the ideal case (ticagrelor 2 × 90 mg, e.g., at 8 a.m. and 8 p.m.) to semichaotic patterns. The latter can be characterized in commonly observed “abrupt lack of adherence” to “morning adherence and no adherence in the evening.”

### Key Risk Factors

Key risk factor data for both visits 1 and 2 for blood pressure and BMI were available for 54.7% and 55.5% of study patients, respectively (Table 2). Few patients contributed data for both visits for levels of LDL cholesterol, HDL cholesterol, and HbA1c (12.6%, 10.1%, and 4.1%, respectively), and these parameters were thus not assessed further. There was little change in blood pressure and BMI between the two visits (Table 2).

**Table 2:** Risk factor values in patients with available data at both visit 1 and visit 2

Risk factor	Active group (*n* = 342)		Control group (*n* = 334)	
	Visit 1	Visit 2	Visit 1	Visit 2
BP, mmHg	*n* = 166		*n* = 158	
Systolic	130.5 (20.7)	127.6 (15.3)	131.3 (22.0)	127.0 (15.2)
	*p*_visits_ = 0.006			
	p^*^ = 0.950			
Diastolic	79.0 (12.6)	78.4 (8.7)	76.9 (14.5)	77.2 (9.6)
	*p*_visits_ = 0.823			
	p^*^ = 0.094			
BMI, kg/m^2^	*n* = 192		*n* = 183	
	28.6 (5.3)	28.4 (4.9)	28.5 (4.6)	28.4 (4.5)
	*p*_visits_ = 0.206			
	p^*^ = 0.803			

Values are shown as mean (standard deviation)

p^*^ calculated between treatment groups (for both visits 1 and 2) using analysis of variance (ANOVA)

*p*_*visits*_, comparisons between visits 1 and 2 (for both the active group and control group) were conducted with the paired *t*-test

*BMI* body mass index, *BP* blood pressure

### HRQoL

SF-36 scores generally indicated an improvement from visit 1 to visit 2 in physical and mental health for both the active (*n* = 131) and control (*n* = 109) groups (Table 3). From visit 1 to visit 2, improvements in SF-36 scores were observed (particularly for physical functioning, vitality, bodily pain, and role limitations due to physical functioning) in both the active and control groups. SF-36 scores were similar between the active and control groups.

**Table 3:** SF-36 scores for physical, emotional and social functioning, role limitations, bodily pain, vitality, and general health perceptions at both visit 1 and visit 2

SF-36 concepts	Active group (*n* = 342) Mean score (SD)		Control group (*n* = 334) Mean score (SD)	
	*n* = 131		*n* = 109	
	Visit 1	Visit 2	Visit 1	Visit 2
Physical functioning	66.3 (24.5)	81.8 (19.8)	66.3 (25.4)	79.7 (19.6)
	*p*_visits_ < 0.001		*p*_visits_ < 0.001	
Physical role functioning	58.9 (27.5)	73.6 (23.2)	62.4 (25.5)	71.3 (24.4)
	*p*_visits_ < 0.001		*p*_visits_ = 0.001	
Emotional role functioning	77.3 (25.7)	77.9 (24.0)	78.9 (26.8)	80.3 (22.7)
	*p*_visits_ = 0.766		*p*_visits_ = 0.574	
Social role functioning	73.0 (27.0)	78.5 (23.8)	77.18 (24.7)	81.1 (22)
	*p*_visits_ = 0.017		*p*_visits_ = 0.077	
Mental health	65.1 (19.3)	71.5 (17.4)	67.43 (19.5)	72.0 (18.4)
	*p*_visits_ < 0.001		*p*_visits_ = 0.003	
Bodily pain	59.4 (29.9)	77.5 (26.8)	58.6 (30.0)	80.1 (23.1)
	*p*_visits_ < 0.001		*p*_visits_ < 0.001	
Vitality	52.3 (21.9)	59.3 (20.0)	54.5 (20.0)	60.3 (18.4)
	*p*_visits_ = 0.001		*p*_visits_ = 0.001	
General health perceptions	61.6 (17.6)	64.7 (19.6)	64.0 (16.5)	64.0 (17.9)
	*p*_visits_ = 0.038		*p*_visits_ = 0.539	

*p*_*visits*_, comparisons between visits 1 and 2 (for both the active group and control group) were conducted with the paired *t*-test

*SD* standard deviation, *SF-36* 36-item Short-Form Health Survey

### Lifestyle Changes

The proportion of patients for whom LSQ data were available at both study visits was 32.7% for healthy eating (active group, *n* = 121; control group, *n* = 100), 33.3% for exercise (active group, *n* = 121; control group, *n* = 104), and 43.6% for smoking (active group, *n* = 153; control group, *n* = 142). At visit 2 compared with visit 1, healthy eating, exercise, and smoking habits showed improvements in the active group, and exercise showed improvements in the control group (Table 4). A slightly bigger increase in patient-reported exercise frequency and smoking cessation was observed in the active group than in the control group from visit 1 to visit 2, although no statistical testing was performed for this comparison.

**Table 4:** LSQ assertions on healthy diet, exercise, and smoking habits at both visit 1 and visit 2

LSQ assertions	Active group (% of patients)		Control group (% of patients)	
	Visit 1	Visit 2	Visit 1	Visit 2
Agreed or partially agreed to a healthy diet	*n* = 266	*n* = 124	*n* = 240	*n* = 101
	85.7	91.9	89.2	82.2
	Visit 1: p^*^ = 0.243 Visit 2: p^*^ = 0.027			
Agreed or partially agreed to exercise regularly	*n* = 265	*n* = 124	*n* = 247	*n* = 104
	52.5	78.2	45.7	64.4
	Visit 1: p^*^ = 0.129 Visit 2: p^*^ = 0.021			
Reported to have quit smoking	*n* = 207	*n* = 86	*n* = 180	*n* = 74
	90.6	91.9	90.6	82.4
	Visit 1: p^*^ = 0.798 Visit 2: p^*^ = 0.072			

p^*^ calculated between treatment groups

*LSQ* Lifestyle Changes Questionnaire

### Disease Understanding, Treatment Awareness, and Healthcare Utilization

The 45 patients in the active group and 33 patients in the control group who reported not having taken their ticagrelor tablet every day at least once during the preceding 4 weeks provided a total of 86 and 55 answers, respectively, on how often they had forgotten to take their tablet and whether they had done so deliberately. Overall, 76.7% and 1.2% of answers in the active group and 70.9% and 7.3% of answers in the control group were that the patient had forgotten to take their tablet “sometimes” or “often,” respectively. Again, among the 78 patients who reported not having taken their ticagrelor tablet every day at least once during the preceding 4 weeks, 89.5% of answers in the active group and 74.6% of answers in the control group were that the patient had “never” done so deliberately. A further 8.1% of answers in the active group and 25.5% in the control group were that they had done so deliberately “sometimes,” and 2.3% in the active group and none (0%) in the control group that they had done so deliberately “often.” Data for BAQ questions 7–15 were available for 402 patients (59.5%; 207 patients in the active group and 195 in the control group) for at least one time point during the study period (Table 5).

**Table 5:** BAQ (Q7–Q15) assertions available for at least one point (of data input) during the study

BAQ assertions	Active group (*n* = 207) (% of patients)	Control group (*n* = 195) (% of patients)	*p* value
Thought it was harmless to not take their ticagrelor tablet (Q7)	39.6	48.2	0.083
Reported never having problems with remembering to take ticagrelor tablet (Q8)	49.8	54.4	0.356
Reported never finding it too inconvenient or difficult to stick to their ticagrelor medication plan (Q9)	58.9	60.5	0.748
Completely or partially understood why they were taking ticagrelor (Q10)	98.1	92.8	0.011
Completely or partially agreed that the good things about taking ticagrelor outweigh the bad (Q11)	88.9	83.6	0.122
Visited a healthcare provider in association with their cardiovascular disease (Q12) [mean number of visits (SD)]	85.5 [6.1 (6.6)]	84.6 [6.3 (6.1)]	0.626
Were admitted to hospital during the study (Q13) [mean number of visits (SD)]	48.3 [1.1 (2.3)]	41.5 [1.4 (3.6)]	0.173
Were admitted to hospital for more than 24 h (Q14)	48.3	41.6	0.173
Were admitted to hospital for more than 24 h on multiple occasions (Q15)	21.5	22.2	0.940

*BAQ* Brilique Adherence Questionnaire, *SD* standard deviation

### App Usage

Among those with data available (171 in the active group and 84 in the control group), a comparable proportion of patients from both groups confirmed their use of an alternative medication reminder or health app (5.3% and 4.8%, respectively). Of patients who assessed the usability of the support tool app, most agreed or strongly agreed that the app was easy to use (78.4% in the active group). A minority of patients in the active group found the app to be unnecessarily complex (14.6% agreed or strongly agreed), with 13.5% agreeing or strongly agreeing that they would need the support of a technical person to be able to use this system. The mean (SD) SUS score for the active group utilizing the support tool app was 74 (20.8), which is considered above average.

## Discussion

Smartphone-based approaches have the potential to improve adherence to drug treatment and healthy lifestyle behaviors in patients with chronic diseases [13–15]. In the current study in patients with ACS prescribed ticagrelor, the delivery of a patient support tool via an electronic device app was associated with improved patient-reported treatment adherence compared with a data collection app alone, without the use of the support tool. At baseline, prevalence patterns for concomitant cardiovascular and metabolic disorders, prior PCI, and concomitant medication use were comparable for the active and control groups, increasing the robustness of the results of this study. Interestingly, there was a bigger increase in patient-reported exercise frequency and smoking cessation in the active group than in the control group from baseline to the end of the study. No notable between-group differences were observed for changes in key cardiovascular risk factors, patients’ disease understanding, treatment awareness, or healthcare utilization during the study. As measured by SF-36, improvements from visit 1 to visit 2 were observed for patient-reported HRQoL and physical and mental functioning measures in both the active and the control groups, with no differences between the groups. Fewer patients conformed to MEMS data input than to BAQ data input. This may be attributed to the increased frequency of input required for MEMS (i.e., twice daily), as opposed to the BAQ (which only required completion once every 4 weeks). Future analysis of MEMS data may offer insight into patient behavioral dosage patterns and help to inform timings of support tool app reminder messages to individual patients.

Patient non-adherence is well recognized worldwide [22]. A lack of awareness regarding the importance of their treatment is a key reason for non-adherence in patients with chronic diseases [23]. This is reflected in the current study; although, overall, more than 90% of responders reported that they understood or partially understood why they were taking ticagrelor, about 45% (39.6% in the active group and 48.2% in the control group) thought it was harmless not to take their ticagrelor sometimes. In its scientific statement on achieving 2020 goals to improve cardiovascular health, the American Heart Association emphasizes the importance of patients having access to, and being able to understand, health information [24]. A survey conducted in Germany has shown that “inadequate” health literacy (defined as ≤ 8/16 points on the HLS-EU-Q16 health literacy questionnaire) was independently associated with the presence of cardiovascular disease and increased healthcare use [25], further affirming the necessity for providing patients with effective information and support to improve health outcomes. Disease management programs (DMPs) set up in 2002 in Germany aim to reduce non-adherence; however, a selection bias may exist, with differences in sex, age, employment status, and comorbidities observed between enrollees and non-enrollees of DMPs [26].

In the current study, patients in the active group received individualized feedback, including optional daily medication adherence reminders and motivational, informative messages every few days, with graphical displays of cardiovascular risks in relation to lifestyle choices, via a support tool app. Patient-reported adherence to ticagrelor treatment during the 48-week observation period was high in both study groups, with mean rates of 96.4% and 91.5% in the active group and control group, respectively. This contrasts with medication adherence rates of 50–60% at 6–24 months reported in other studies of patients with cardiovascular disease [8, 9]. In a US registry study that included 6434 patients with acute MI, the rate of patients’ self-reported persistence with prescribed medications was 64% at 1 month, 58% at 6 months, and 57% at 12 months after discharge [9]. A meta-analysis of data on 376,162 patients with cardiovascular diseases across 20 studies demonstrated an adherence rate of 57% after a median of 24 months, estimated based on prescription refills [8].

Only 50.9% of patients in the active group and 52.1% of those in the control group completed the BAQ, which provided the primary endpoint (adherence) data. The results are limited by this relatively low response rate to questionnaires across both groups. It is likely due to the true real-world nature of this study, with no actual study visits or other communication with the patients besides the tool itself. The need to make this a true real-world study was prioritized to keep the intervention to only the technology and avoid all other aspects. Although the lower than expected response rate hampers the generalizability of the corresponding results, we deduce that it is plausible that the patients who responded to the questionnaires during the study were generally diligent, and thus also likely to adhere to their treatment. This could explain the high adherence rates in both study groups. If this was indeed the case, then the fact that there was still a significant (albeit small) difference in adherence between groups is particularly noteworthy. Taking into account the low cost and easy scalability of providing app tools for patients, even small improvements in adherence should be considered a success, with the potential for important impacts on health outcomes and healthcare costs at both the patient and the population level. This study revealed a high patient satisfaction rate with the support tool app (in the active group) with a SUS score of 74 (68 is considered average [27]). A similar study utilizing an interactive support app also reported high patient satisfaction rates, with a SUS score of 87 within the active group [13].

No on-site visits were included in the 48-week observation period of this study, and PROs were assessed by questionnaires that patients completed remotely on their own devices and in their own setting. This design places the study in a real-world setting. Contact with study healthcare professionals was thus minimized to just two clinic visits, one at baseline and one at the end of the study (i.e., 12 months after the ACS event). An additional on-site visit may have helped to improve the response rate.

In a recent study of patients with coronary heart disease, a significant difference in medication adherence was observed between reminder app users and a usual care group, assessed using the Morisky Medication Adherence Scale (MMAS-8) [14]. The mean MMAS-8 score at the 3-month follow-up (the primary endpoint) was 7.1 in the app user group, compared with 6.6 in the control group (*p* = 0.008) [14]. Interestingly, providing patients with an advanced app with additional interactive and customizable features did not result in improved MMAS-8 scores compared with the basic app [14]. Unlike the MMAS-8 data, no differences between the groups were observed when adherence was assessed via the number of pills missed in the past 7 days [14]. The MMAS-8 captures medication-taking behaviors in general rather than adherence specifically [28] and was thus not considered “fit for purpose” for the current study [17]. Furthermore, Johnston et al. showed that the use of an interactive support app increased treatment adherence compared with a basic app in patients with MI [13]; adherence was a composite study endpoint defined by a combination of non-adherence and treatment gaps of more than four doses [13]. The number of events of non-adherence was lower in the active group than in the control group (16.6 events vs 22.8 events; *p* = 0.025) over 6 months, and differences in lifestyle changes were not statistically significant [13]. The results of such studies and the current study support the use of an interactive support app for increasing patient adherence.

There were some further limitations of the current study. Most notably, as already discussed, only about half of patients provided questionnaire data for the primary study outcome, and even fewer patients provided data for most secondary endpoints. Eighty-eight patients were lost to follow-up. Patients were asked about their adherence in the past 7 days only at 4-week intervals, with results being extrapolated to the past 4-week period. While this approach minimized the risk of patients’ adherence behaviors being affected by the process of adherence measurement and lessened recall bias, it may have affected the accuracy of the results. The BAQ and LSQ were developed specifically for this study, and their validity and reliability will need to be evaluated in future analyses. Using the same tool for intervention and data collection may have affected patients’ reporting behaviors.

Educational level, living arrangements, and employment status were balanced between the active and control groups, and their impact on the results should thus be minimal. A recent systematic review and meta-analysis on the prevalence and predictors of medication non-adherence assessed demographic variables, including age, sex, marital status, education, and deprivation, and concluded that no demographic variables significantly predicted non-adherence [29].

In conclusion, the delivery of a patient support tool via an electronic device app was associated with improved patient-reported treatment adherence compared with a data collection app alone in patients prescribed ticagrelor for ACS. Positive trends in association with the support tool app were also observed for patient-reported exercise frequency and smoking cessation.

## Supplementary Information

Below is the link to the electronic supplementary material.

Supplementary file1 (DOCX 72 KB)

## Data Availability

Not applicable.
