# Supplementary material for: Results from the “Me & My Heart” (eMocial) Study: a Randomized Evaluation of a New Smartphone-Based Support Tool to Increase Therapy Adherence of Patients with Acute Coronary Syndrome
Source: Cardiovasc Drugs Ther. 2022 Apr 20;37(4):729–41. doi: 10.1007/s10557-022-07331-1 (PMC10397150; doi:10.1007/s10557-022-07331-1)
Supplement: Supplementary file 1 — Supplementary file1 (DOCX 72 KB) [file 10557_2022_7331_MOESM1_ESM.docx]

# Results From the “Me & My Heart” (eMocial) Study: a Randomized Evaluation of a New Smartphone-Based Support Tool to Increase Therapy Adherence of Patients with Acute Coronary Syndrome

**Journal name**

*Clinical Research in Cardiology*

**Author names**

Florian Krackhardt • Magnus Jörnten-Karlsson^2^• Matthias Waliszewski • Mikael Knutsson • Anna Niklasson • Karl-Friedrich Appel • Ralf Degenhardt • Alexander Ghanem • Till Köhler • Marc-Alexander Ohlow • Carsten Tschöpe • Heinz Theres • Jürgen vom Dahl • Björn W. Karlson • Lars S. Maier

**Affiliation and e-mail address of corresponding author**

Florian Krackhardt, MD, Department of Internal Medicine and Cardiology, Charité Campus Virchow-Klinikum, Charité University Medicine Berlin, Augustenburger Platz 1, 13353 Berlin, Germany. E-mail: [florian.krackhardt@charite.de](mailto:florian.krackhardt@charite.de)


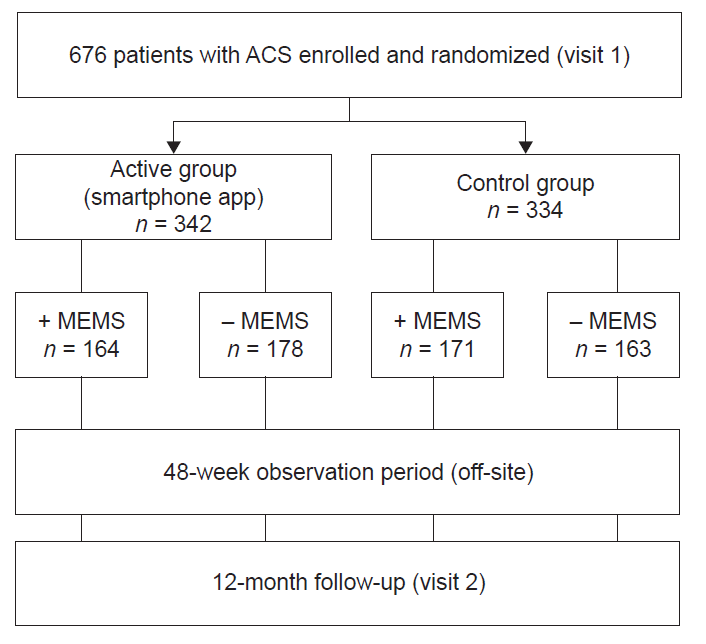


**Supplementary Fig. 1** Study flow chart. *ACS* acute coronary syndrome, *MEMS* Medication Event Monitoring System

## Supplementary Table 1 Support tool app

| **Components** | **Details** |
| --- | --- |
| Education about disease and lifestyle habits | - Information on disease, importance of treatment, and support to create new lifestyle habits |
| DAPT medication management | - Reminders to take medication twice per day - Patient-recorded information on when medication was taken - Reminders for prescription refill |
| Motivational messages | - Personalized based on time since ACS, patients’ targets, and other patient-recorded information, to promote healthy behaviors and lifestyle changes |
| Biometrics | - Biometrics such as blood pressure, blood lipids, and blood glucose (patient-recorded) to help illustrate key parameters |
| Optional target-setting | - Targets for exercise level, body weight reduction (if BMI > 25 kg/m^2^), and smoking cessation/reduction |
| Treatment index | - Weekly overview of patients’ treatment adherence and lifestyle habits, to applaud and motivate |

*ACS* acute coronary syndrome; *BMI* body mass index; *DAPT* dual antiplatelet therapy*.*

## Supplementary Table 2 Brilique Adherence Questionnaire (BAQ)

| Effect on adherence | |
| --- | --- |
| 1 | Do you currently take ticagrelor? |
| 2 | If not, why are you not taking ticagrelor? |
| 3 | Over the past 4 weeks, did you take your ticagrelor tablets every day? (If ‘yes’ go directly to Q7) |
| 4 | For patients who did not take all ticagrelor tablets every day, how many ticagrelor tablets did you take during the last 7 days? |
| Effect on disease understanding and treatment awareness | |
| 5 | How often did you forget to take your ticagrelor tablets? |
| 6 | Over the past 4 weeks, how often did you deliberately not take your ticagrelor tablets? |
| 7 | Do you think it is harmless if you sometimes do not take your ticagrelor tablets? |
| 8 | Over the past 4 weeks, did you ever have problems remembering to take your ticagrelor tablets? |
| 9 | Do you find it inconvenient or difficult sticking to your ticagrelor medication plan? |
| 10 | Do you understand why you are taking ticagrelor? |
| 11 | Do you think that the good things about taking ticagrelor outweigh the bad? |
| Effect on healthcare utilization | |
| 12 | Have you made any visits associated with your cardiovascular disease to your healthcare provider without being admitted to hospital? |
| 13 | How many visits have you had with your healthcare provider? |
| 14 | Have you at any time been admitted to hospital for more than 24 hours? |
| 15 | How many times have you been admitted to hospital for more than 24 hours? |

**Supplementary Table 3** Baseline educational level, living arrangements and employment status.

| Variable | Active group (*n* = 342) | | Control group (*n* = 334) | *p* value |
| --- | --- | --- | --- | --- |
| Education level, *n* (%) |  | |  | 0.838 |
| No degree | 4 (1.2) | | 4 (1.2) |  |
| Secondary school | 175 (51.2) | | 177 (53.0) |  |
| Technical college | 59 (17.3) | | 48 (14.4) |  |
| University | 44 (12.9) | | 40 (12.0) |  |
| Unknown | 60 (17.5) | | 65 (19.5) |  |
| Living with a partner, *n* (%) |  |  | |  |
| Yes | 188 (55.0) | 164 (49.1) | | 0.127 |
| No | 154 (45.0) | 170 (50.9) | |  |
| Employed or self-employed, *n* (%) |  | |  | 0.348 |
| Yes | 230 (67.3) | | 214 (64.1) |  |
| No | 112 (32.7) | | 120 (35.9) |  |

*p* values calculated with the Pearson’s chi-square test.
